# Cerebrospinal fluid lens-free microscopy: a new tool for the laboratory diagnosis of meningitis

**DOI:** 10.1038/srep39893

**Published:** 2017-01-03

**Authors:** Robin Delacroix, Sophie Nhu An Morel, Lionel Hervé, Thomas Bordy, Jean-Marc Dinten, Michel Drancourt, Cédric Allier

**Affiliations:** 1 Aix Marseille Univ, INSERM, CNRS, IRD, URMITE, Marseille, France; 2 Université de Grenoble Alpes, F-38000 Grenoble, France, CEA, LETI, MINATEC Campus, Technologies for Healthcare and Biology division, F-38054 Grenoble, France

## Abstract

Cerebrospinal fluid cytology is performed by operator-dependant light microscopy as part of the routine laboratory work-flow diagnosis of meningitis. We evaluated operator-independent lens-free microscopy numeration of erythrocytes and leukocytes for the cytological diagnosis of meningitis. In a first step, prospective optical microscopy counts of leukocytes done by five different operators yielded an overall 16.7% misclassification of 72 cerebrospinal fluid specimens in meningitis/non-meningitis categories using a 10 leukocyte/μL cut-off. In a second step, the lens-free microscopy algorithm adapted for counting cerebrospinal fluid cells and discriminating leukocytes from erythrocytes was modified step-by-step in the prospective analysis of 215 cerebrospinal fluid specimens. The definite algorithm yielded a 100% sensitivity and a 86% specificity compared to confirmed diagnostics. In a third step, a blind lens-free microscopic analysis of 116 cerebrospinal fluid specimens, including six cases of microbiology-confirmed infectious meningitis, yielded a 100% sensitivity and a 79% specificity. Adapted lens-free microscopy is thus emerging as an operator-independent technique for the rapid numeration of leukocytes and erythrocytes in cerebrospinal fluid. In particular, this technique is well suited to the rapid diagnosis of meningitis at point-of-care laboratories.

Performing the cytological analysis of the cerebrospinal fluid (CFS) and enumerating leukocytes and erythrocytes is a routine first step in the laboratory diagnosis of meningitis^1^,^2^,^3^,^4^. Indeed, meningitis is diagnosed if more than 10 leukocytes/μL are counted, in the absence of erythrocytes^5^,^6^. CSF cytology and cell counting is routinely performed by optical microscopy. Optical microscopy observation is an operator-dependant task, with both counting itself and the subsequent reporting being subject to variability; this may indeed result in the erroneous classification of the CSF specimen as meningitis/non-meningitis. Accordingly, optical microscopy cytological analysis of the CSF can hardly be incorporated into the point-of-care (POC) laboratory for the rapid diagnosis of meningitis^7^,^8^.

Therefore, we aimed to develop an alternative, operator-independent method for counting CSF leukocytes and erythrocytes, which would require a CSF volume <50 μL, and would be suitable for connection to the laboratory’s information systems and thus, which would be better adapted to the POC laboratory^7^. A few automatic methods have been shown to accurately count leukocytes and erythrocytes in CSF, but they are ill-suited to the POC because of their size and complexity^9^. We considered that lens-free microscopy, an emerging microscopy technique based on in-line holography^10^, could be an option. In lens-free microscopy, objects are illuminated by a light plane wave, and a complementary metal oxide semi-conductor (CMOS) sensor records the holographic pattern resulting from the interference between the light diffracted by the micrometric structures and the incident wave. This setup allows one to acquire, at glance, a large field-of-view of ~30 mm^2^ and the image of every single cell present in the sample can be retrieved through the computation of a phase retrieval algorithm^11^,^12^. Lens-free microscopy thus appears to provide a particularly promising technique for diagnostic imaging at the POC laboratory, including low-resource settings^13^,^14^,^15^.

We thus evaluated the ability of lens-free microscopy to perform CSF cytology to contribute to establishing an early detection of infectious meningitis, in three successive steps. In the first step, we studied the inter-individual variability of CSF cytology performed by the reference standard optical microscopy. In the second step, we set-up a lens-free microscopy algorithm adapted for counting CSF cells and capable of discriminating leukocytes from erythrocytes based on the prospective analysis of 215 CSF specimens. In the third step, we established a proof-of-concept that lens-free microscopy and the algorithm created allowed for the rapid cytology-based laboratory diagnosis of meningitis. This proof-of-concept has been demonstrated through the blind assay of 116 further CSF specimens.

## Results

In the first step, the inter-operator variability of optic-microscopy was assessed on 72 CSF samples that were prospectively, independently, and blindly observed by five different operators using optical microscopy (Supplementary Table 1). Of the 72 samples analyzed, inter-operator agreement (≥3/5 operator agreement) yielded 28 CSF specimens with a leukocyte value ≥10 leukocytes, classifying them as meningitis. In this 72-CSF specimen series, 12 CSF specimens (16.7%) were misclassified by at least one operator. More precisely, of 44 non-meningitis CSF samples, (leukocytes <10), nine specimens (20.5%) were misclassified as meningitis by at least one operator. Of the 28 meningitis CSF (leukocytes ≥10), three specimens (10.7%) were misclassified as non-meningitis by at least one operator. Although limited in time and in the number of CSF specimens analyzed, this study nevertheless illustrated the significant inter-individual operator variability of CSF cell counting using standard optical microscopy.

**Figure 1:**
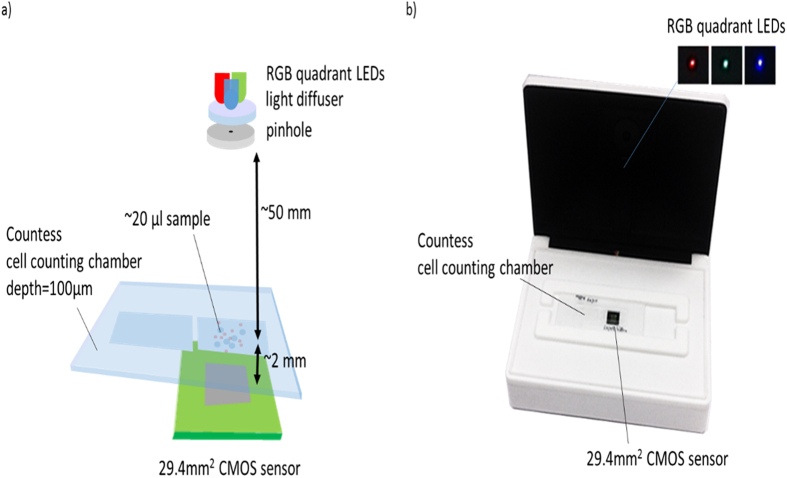
Experimental setup for the lens-free analysis of CSF clinical specimen. (**a**) Schematic of the optical setup. The multispectral illumination is provided by a multi-quadrant RGB LED coupled to a 150 μm pinhole through a 60° diffuser. When the quadrant RGB LED sequentially illuminates the sample, the CMOS sensor records three red, green and blue holograms. (**b**) Picture of the prototype used in the proof of concept study. The device cover featuring the RGB illumination is shown open in the picture.

**Figure 2:**
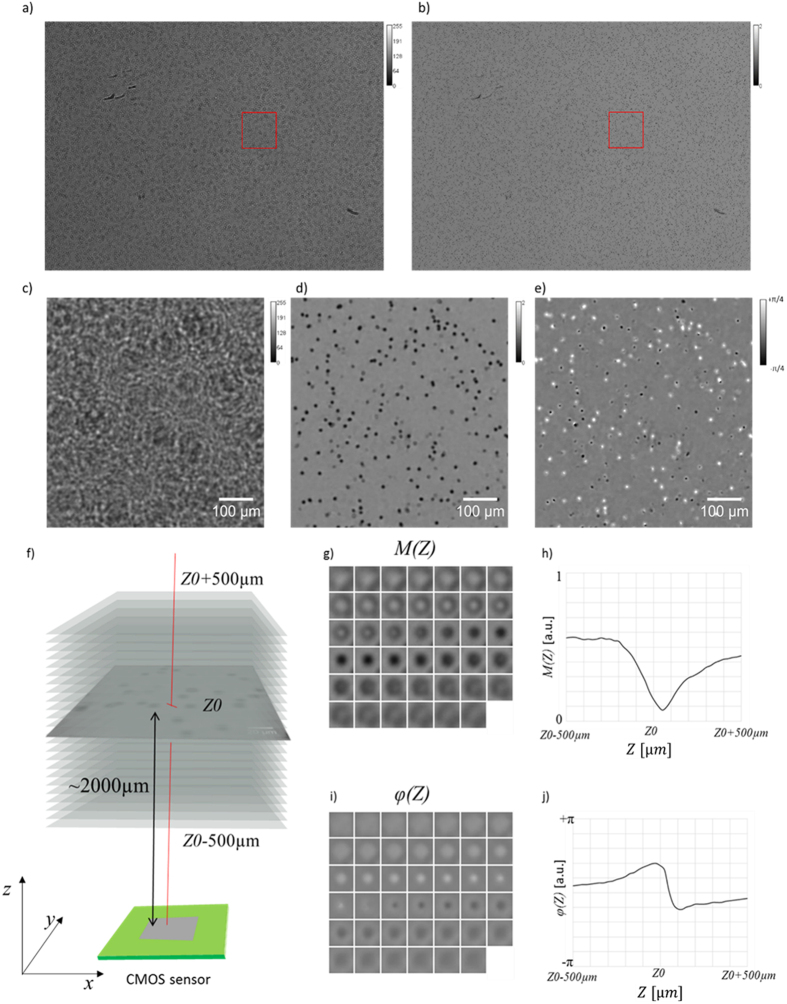
(**a**) Lens-free raw wide-field hologram acquired in the blue channel by means of a CMOS image sensor (field of view of ~29.4 mm^2^) of the sample Q160546732 (ruptured brain aneurysm; microscope counting: 6400 erythrocytes/μL, 40 leukocytes/μL). (**b**) Lens-free reconstructed module image in the blue channel. (**c**,**d**) Are details of respectively (**a**,**b**). (**e**) Detail of the lens-free reconstructed phase image in the blue channel. (**f**) The propagation of the reconstructed image by either side of the object plane over 1000 μm at 20 μm intervals results in stacks of 41 complex images (not to scale). (**g**,**i**) Montage of the module and phase stack respectively centered on a cell in particular. The dimension of the images are 24 * 24 μm^2^. (**h**,**j**) Module and phase and *Z*-axis profiles measured in the center of the cell stack images (**g**,**i**) as a function of the distance Z to the sensor plane. Z0 corresponds to the distance from the sensor plane to the cell.

**Figure 3:**
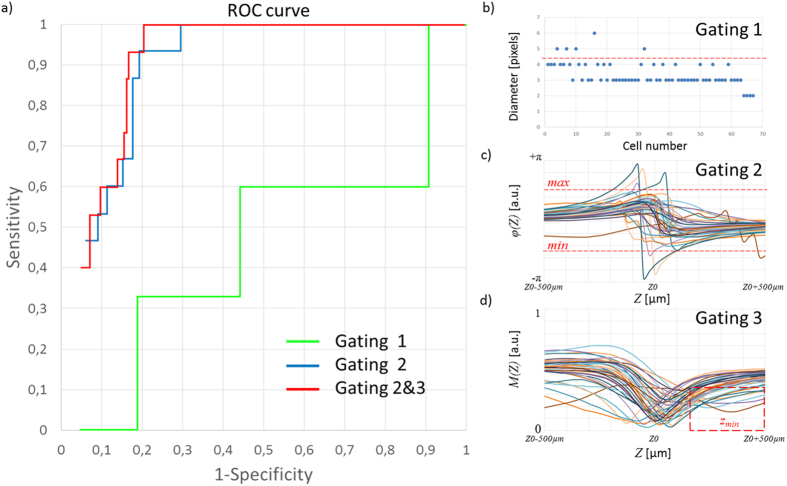
(**a**) Receiver operating characteristic (ROC) curves obtained with different lens-free classification algorithm for the detection of infectious meningitis. The green curve is obtained with a leukocytes count based on the measurement of the cell diameter. This gating is depicted in (**b**), a threshold (red dotted line) is applied to the distribution of the cell diameter in pixels (1.67 μm pixel pitch). This threshold has been varied to compute the green ROC curve in (**a**). The blue ROC curve in (**a**) corresponds to the classification obtained with a gating applied to the phase Z-axis profiles measurement (Fig. 2h). This gating is depicted in (**c**). It selects phase Z-axis profiles of which the amplitude is larger than a defined threshold (red dotted line). The red ROC curve in (**a**) corresponds to the classification obtained with the phase gating and a module gating. The module gating is depicted in (**d**). It selects module Z-axis profiles (Fig. 2j) of which the local minima Zmin is well above the Z0.

**Figure 4:**
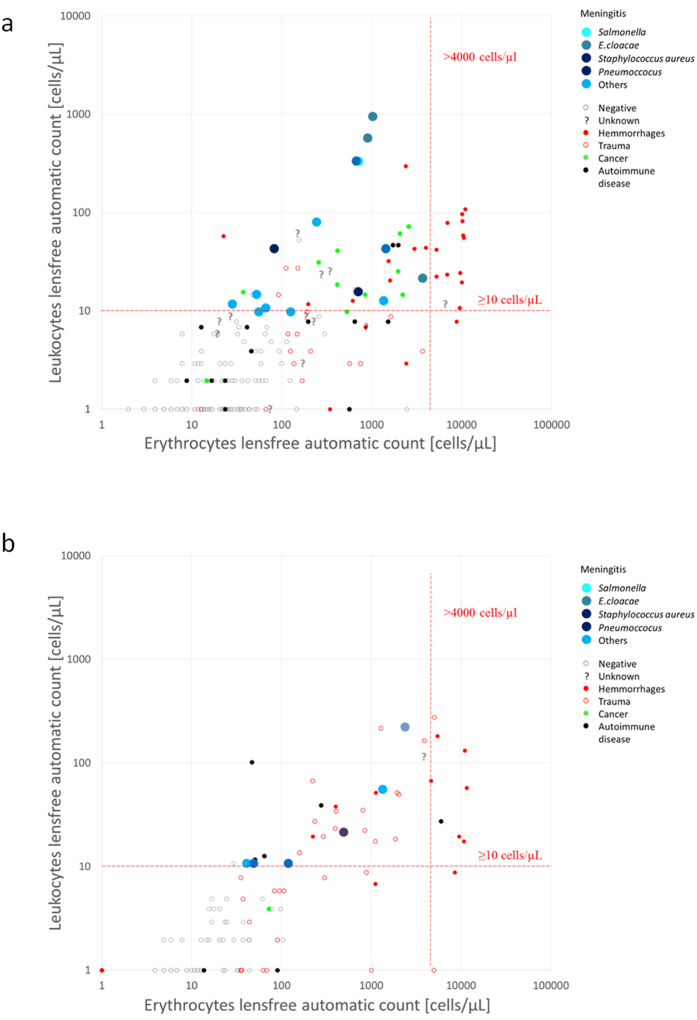
(**a**) Scatterplot of the automatic lens-free count of leukocytes and erythrocytes resulting from the analysis of the first datasets featuring 215 clinical specimens. A color code has been defined with respect to the different diagnosis established for all clinical specimens. The infectious meningitis of interest are plotted in large blue dots. The limit of 10 leukocytes cells/μl used for the biological definition of meningitis is depicted by a horizontal red dotted line. A second limit set on the number of erythrocytes of 4,000 cells/μL allows to further discard false positives that can be considered as hemorrhages. It is depicted by a vertical red dotted line. Using this additional criterion, lens-free microscopy achieved a sensitivity of 100% and a specificity of 86%. (**b**) Scatterplot of the automatic lens-free count of leukocytes and erythrocytes resulting from the analysis of the first datasets featuring 116 clinical specimen used in the proof-of-concept study. We counted in total 31 positive samples corresponding to six true positive of infectious meningitis and 25 false positives cases. Eight false positives diagnosed are further discarded by applying additional criteria on the on the number of erythrocytes. As a result, lens-free microscopy achieved a sensitivity of 100% and a specificity of 79% for the diagnosis of infectious meningitis.

Based on the observation of inter-operator variability in optical microscopy counting of CSF cells, we aimed to progressively adapt the previous versions of lens-free microscopy and algorithms^16^,^17^ for use in the detection of leukocytes and erythrocytes in CSF. In this second step of our study, we analyzed a dataset of 215 CSF specimens and, step-by-step, we modified the lens-free microscopy protocol and its associated algorithms. This dataset of 215 CSF clinical specimens included 15 cases of microbiology-confirmed infectious meningitis and 48 cases of non-infectious meningitis, including 11 cases of carcinomatous meningitis, 24 cases of hemorrhages, and 13 cases of autoimmune diseases. The dataset also featured 140 negative cases among which 16 samples presented a large number of blood cells consecutive to a traumatic lumbar puncture. The remainder included 12 cases (6%) of which the diagnosis was not deemed definitive at the time of the study. Lens-free microscopy acquisitions were performed at three different wavelengths in order quickly and efficiently to reconstruct the image of the cells with a resolution sufficient to obtain size and position information (Fig. 1). An optical signature for every cell was obtained by propagating the reconstructed complex image of the cell along the optical axis at different distances *Z* from the sensor plane (Fig. 2). Based on this optical signature, we then developed an algorithm for counting CSF leukocytes and erythrocytes in CSF samples, to establish an early diagnosis of meningitis. On the basis of this first CSF dataset, we established an algorithm to detect CSF specimens with a leukocyte concentration >10 cells/μL, i.e. the limit used for the biological definition of meningitis. Cell counting by microscopic counting, with the reading done by an operator, was performed for comparison. Figure 3 shows the receiver operating characteristic (ROC) curves obtained with different lens-free classification algorithms. The first lens-free classification algorithm performs a gating on the measured cell diameter to count the leukocytes and test the limit of 10 leukocytes/μL. The results are not convincing, as they are close to the threshold of non-discrimination. Accordingly, the reconstructed module image presented an insufficient resolution to distinguish the different blood cells based on size criteria, i.e. erythrocytes with a disk diameter of approximately 6–8 μm and leukocytes measuring 7–30 μm in diameter. In particular, the distinction between the erythrocytes and smaller lymphocytes, measuring 7–8 μm in diameter was critical. In order to improve the lens-free discrimination between leukocytes and erythrocytes, we further applied gating to the reconstructed phase and module in the *Z*-axis profile (Fig. 3). To detect the leukocytes, a threshold was applied to the profile amplitude, i.e. the difference between the local maxima and minima (Fig. 3c). We assumed that the leukocytes presented a larger phase shift than erythrocytes owing to the difference in size. This assumption was confirmed over a limited number of cells by comparing the lens-free results with observations under microscope (Fig. S1). With a threshold applied at 1.38 radians (80 degrees), the sensitivity increased to 100%, and the specificity to 70% (Fig. 3a). This gating is performed without any phase unwrapping. Phase discontinuities exist (Fig. 3c, Fig. S1c,e) and lead to leukocytes detection since the phase profile amplitude is then obviously larger than the threshold set to 1.38 radians. With a second gating applied to the module *Z*-axis profiles (Fig. 3d), the specificity increased to 80% (Fig. 3a). This second gating was intended to detect the outlier profiles which may correspond to granular leukocytes, which were in fewer numbers than lymphocytes. These results have been obtained with the cell detection performed using the blue channel (450–465 nm) and the detection of leukocytes using the red channel (620–630 nm). For comparison, if both detection and analysis are performed in the red channel, the specificity decreases down to 71.5%. And the obtained specificity is even lower than 65%, if we use the blue channel or the green channel only. Hence it appears that the blue channel allows one to perform the best cell detection while the red channel allows to obtain the best leukocytes detection. Figure S2 shows the comparison between the automatic lens-free count and the microscopic manual count. We observed a linear correlation between the lens-free and optic-microscopy counts up to concentrations of ~5.10^4 ^cells/μL. For the leukocyte count, the results between the lens-free and the microscope are less correlated, as lens-free microscopy systematically overestimated the leukocyte count compared to the optic-microscopy count. Two cases of infectious meningitis which remained undetected under microscope (with PCR-based detection of CSF pathogen), in which the leukocyte optic-microscopy count was, respectively, six and seven leukocytes/μL, were well below the limit of 10 cells/μL used for the early diagnoses of meningitis (Fig. S3). These two samples were positively detected by our algorithm, with leukocyte counts of 10 and 21 leukocytes/μL, respectively. Figure 4a shows the scatterplot of the lens-free count of leukocytes and erythrocytes for these 215 clinical specimens, corresponding to the best classification obtained with two gating steps (Fig. 3a). In total we counted 57 positive samples corresponding to 15 true positives for infectious meningitis, and 42 false positives. Among the false positives, we count 20 cases of hemorrhages, nine cases diagnosed with carcinomatous meningitis (linked to glioma, medulloblastoma and carcinoma), and two cases diagnosed with autoimmune disease. Remaining were three cases of trauma and four cases of meningitis of unknown etiology. False positives diagnosed as hemorrhages were further discarded by applying additional criteria to the number of erythrocytes, set to 4,000 cells/μL (Fig. 4). Using the additional criteria, the new lens-free protocol and algorithm achieved a sensitivity of 100% and a specificity of 86%.

In a third step, we measured the performance of lens-free microscopy and an algorithm for the diagnosis of infectious meningitis. In a blind assay, we prospectively compared lens-free microscopy on 116 CSF clinical specimens with the diagnosis actually determined for every patient. The results for meningitis/non-meningitis categories using a 10 leukocyte/μL cut-off allow one to establish the sensitivity and specificity of lens-free microscopy. The dataset of 116 CSF clinical specimens included six cases of microbiology-confirmed infectious meningitis, and 20 cases of non-infectious meningitis, including four cases of carcinomatous meningitis, 12 cases of hemorrhages, and four cases of autoimmune diseases. The dataset also featured 90 negative cases, among which 31 samples presented a large number of blood cells consecutive to a traumatic lumbar puncture. In the one remaining case, the diagnosis was not definitive at the time of the study. Figure 4b shows the scatterplot of the lens-free count of leukocytes and erythrocytes for these 116 clinical specimens, corresponding to the best classification obtained with the two gating steps previously defined (Fig. 3). In total we counted 31 positive samples corresponding to six true positive with infectious meningitis and 25 false positive cases. Among the false positives, we counted nine cases of hemorrhages, two cases diagnosed with cancer (glioma, medulloblastoma, and carcinomatous cancer) and three cases diagnosed with autoimmune diseases. The remaining were 15 cases of trauma and one cases of meningitis of unknown etiology. Eight false positives diagnosed as hemorrhages were further discarded by applying additional criteria with the number of erythrocytes set to 4,000 cells/μL (Fig. 4b). Using the additional criteria, the new lens-free protocol and algorithm achieved a sensitivity of 100% and a specificity of 79% for the diagnosis of infectious meningitis.

## Discussion and Conclusion

The various existing lens-free microscopy techniques able to count leukocytes and erythrocytes cannot usefully be applied to cell counts in CSF. The first method consists in analyzing the holographic pattern acquired by the CMOS sensor^15^,^16^ without reconstructing the image of the cells. This is a very efficient means for the classification of the blood cells. Leukocytes subpopulations can be distinguished, i.e. neutrophils, lymphocytes, and monocytes. Unfortunately this method can only be applied to samples with low a concentration of cells, or diluted samples, to ensure there is no overlap in the sensor plane between the cells’ holographic signatures. Yet the expected cell concentration of CSF spans several orders of magnitude, from a few cells per μL up to 10^6 ^cells per μL. In order to analyze low and high concentrations in clinical specimens, it was necessary to reconstruct the image of the cells with a dedicated holographic reconstruction algorithm. However, low-resolution images of the blood cells (pixel size = 1–2 μm, spatial resolution >2 μm) prevented an automated analysis based on image processing. It was known that sub-pixel super-resolution techniques could be applied to improve the spatial resolution down to ~1 μm^17^. Accordingly, lens-free microscopy in combination with sub-pixel super-resolution was successfully applied to the imaging of smears and tissue slides^18^,^19^,^20^. Such a pixel super-resolution technique requires the collection of a large number of lens-free acquisitions (>50) on a fixed sample, which is not the situation as for the CSF. Here, we established the proof-of-concept that an adapted lens-free microscopy protocol could be used in the laboratory instead of the manual cell-counting diagnosis of meningitis. The CSF cell count method using the lens-free technology has the same level of reliability as optical microscopy, though it possesses greater reproducibility, with a significant time-saving capacity. This method also has another advantage, that of being applicable in the POC laboratory and “in low-resource settings,” unlike previously developed apparatus^9^. Lens-free microscopy applied to CSF will be amenable to future developments, including distinguishing polymorphonuclear leukocytes and the direct detection of pathogens in order to further refine the laboratory diagnosis of CSF diseases, including procedures conducted in the POC laboratory.

## Methods

CSF leftovers were used, so that no additional sampling was specifically done for this study, which was approved by the *Institut Fédératif de Recherches* 48, Marseille.

### Inter-operator variability in CSF cytology

A prospective blind inter-operator variability study was conducted for two months. It consisted of optical microscopy cell-counting conducted by five different operators on 35 consecutive and independent CSF specimens. The five operators were laboratory technicians and residents in medical biology. Each operator manually registered the count results in a table that was not accessible to the other operators. The cell counts were performed on KOVA Glasstic 10 counting slides with grids (Garden Grove, USA), performing the count of leukocytes and erythrocytes in the nine cell segmentations corresponding to the count per mm^3^. The gold-standard working volume of CSF is 10 μL and 6.6 μL are deposited by capillarity into the counting slide. The optical microscope used in the study was an Olympus CX41 (USA), with a magnification of ×40 to ×100. The results of the different counts for each of the CSF specimens were registered in a dataset by an independent operator. The individual variability was determined by a comparative statistical analysis of ANOVA type of variations.

### Implementation of lens-free microscopy and an algorithm adapted to CSF cytology

A total of 215 CSF specimens were prospectively analyzed in order to optimize the lens-free microscopy acquisition and algorithm. Our system was based on lens-free microscopy with the optical setup described by Ozcan *et al*.^16^, which we modified so to perform multi-wavelength acquisitions on CSF clinical specimens. At first, 20 μL of the CSF specimen was injected in a 0.1 mm thick Countess cell counting chamber, and the chamber was put directly on top of the CMOS sensor. As illustrated in Fig. 1, a wide-field hologram acquisition was performed with a 3840 * 2748 pixel monochromatic CMOS image sensor. The sensor pixel size was 1.67 μm, and the field of view was 6.4 mm * 4.6 mm = 29.44 mm^2^. Hence the volume of CSF specimen analyzed with one lensfree acquisition is about 3 μl (29.44 mm^2^  * 0.1 mm). The Countess cell counting device features two chambers, allowing duplication of the reading if necessary. The illumination source was about 5 cm away from the chamber, and was based on a Cree MC-E Color quadrant RGB LED emitting respectively at 620–630 nm, 520–535 nm, and 450–465 nm. This was coupled to a 150 μm pinhole through a 60° diffuser. The distance between the quadrant RGB LED and the diffuser was approximately 2 mm, in order to merge the three colored lights into a single-point source. The acquisition was sequential, and each red, green and blue LED was lit up one after the other, while the three corresponding wide-field holograms were acquired by the CMOS sensor (red, green, and blue). The lens-free optical setup did not acquire magnified images of the cells, but rather, jus the holographic interference patterns from the cells. It is therefore necessary to use a holographic reconstruction algorithm to recover the phase and module images of the cells. The holographic reconstruction consists first in back-propagating the light intensity as recorded by the sensor using the Fresnel function. The recovered complex image, however, is strongly affected by the so-called ‘twin image’, an artefact that results from a lack of phase information in the acquisition process. To retrieve the phase in the sensor plane and consequently diminish the ‘twin-image’ artefact in the reconstructed images, we used a phase-retrieval algorithm. The purpose of such an algorithm is to recover the complex image of the sample from the phaseless recorded holographic image^11^. We developed such an algorithm based on the formulation of the phase retrieval problem discussed in ref. 12. The algorithm is detailed in the supplement (Figs S4 and S5). It allows us to refine the estimate of the complex image in the sensor plane by iteratively applying update rules in a gradient descent scheme. Figure 2 shows an example of the reconstruction image with the amplitude and phase obtained from the acquisition of a CSF specimen rich in red blood cells (>2,000 cells/μL, microscopic count) and presenting sparse white blood cells (<100/μL) as determined using the reference method, i.e., manual counts with a binocular microscope. Thus it was possible to observe simultaneously a large number of cells with a spatial resolution sufficient to count the number of cells, determine their centroids, and measure their approximated diameter. The cell segmentation was performed on the reconstructed red module image (Fig. S6). It was based on a Hough transform, which can efficiently detect the circle patterns present in the image. To improve the detection efficiency, the reconstructed module image was defocused by Δz~250 μm, which improved the contrast of the cells’ circular patterns. Next, in the centroid of all detected objects, the module and phase information were reconstructed as a function of the distance z from the sensor plane (Fig. 2f–j). These complex *Z*-axis profiles were calculated on either side of the object plane over 1000 μm at 20 μm intervals, and provided an optical signature for every single cell present in the CSF specimen. Now the analysis of these *Z*-axis profiles allowed us to discriminate efficiently between the cells and other micro-objects, e.g. particles present in the CSF specimen, or at the surface of the fluidic chamber. Next, a second algorithm would perform the automatic count on the leukocytes and erythrocytes present in the CSF clinical specimen to establish an early diagnosis of meningitis. These calculations can be performed on a laptop computer (CPU Core i7 2.7 GHz, GPU M200M 4Go) at a speed of ~35 seconds per analysis based thanks to a GPU implementation, which is fast enough for a point of care application.

### Proof-of-concept study

In order to establish the proof-of-concept, 116 lens-free additional acquisitions were prospectively performed using the method developed in the second step of this study. CSF cytology was performed in parallel by optical microscopy and by lens-free microscopy using the algorithm previously developed. These CSF specimens were collected in cases of clinical suspicion of meningitis as a part of routine work-flow of the laboratory. All specimens were unique, and differed from those used in the second step of this study. The algorithm developed through acquisitions in the second part of the study was applied to these acquisitions, without being modified. After the processing the acquisitions using the software, the results for meningitis/non-meningitis categories using a 10 leukocyte/μL cut-off were compared to the actual diagnosis determined for every patient. We then determined the sensitivity and specificity of the lens-free microscopy.

## Additional Information

**How to cite this article**: Delacroix, R. *et al*. Cerebrospinal fluid lens-free microscopy: a new tool for the laboratory diagnosis of meningitis. *Sci. Rep.* **7**, 39893; doi: 10.1038/srep39893 (2017).

**Publisher's note:** Springer Nature remains neutral with regard to jurisdictional claims in published maps and institutional affiliations.

## Supplementary Material

Supplementary Information

Supplementary Table 1
