# Supplementary material for: Cerebrospinal fluid lens-free microscopy: a new tool for the laboratory diagnosis of meningitis
Source: Sci Rep. 2017 Jan 3;7:39893. doi: 10.1038/srep39893 (PMC5206666; doi:10.1038/srep39893)

Cerebrospinal fluid lensfree microscopy: a new tool for the laboratory diagnosis of meningitis

Robin Delacroix[a], Sophie Nhu An Morel[b], Lionel Hervé[b], Thomas Bordy[b], Jean-Marc Dinten[b], Michel Drancourt[a]*, Cédric Allier[b]

SUPPLEMENTARY INFORMATION

SUPPLEMENTARY INFORMATION TEXT

Phase-retrieval holographic reconstruction

Figure S4 presents a scheme of our lens-free in-line holography configuration and introduces the notations represents. An incident plane wave U_{inc} illuminates an object with complex transmission α_0, located at plane *z=0*. The complex amplitude in the plane right after the object is expressed as:

$$A_0 = U_{inc}.\alpha_0 \qquad (1)$$

In the sensor plane located at plane $z = Z$, the complex amplitude A_Z results from the convolution of the complex amplitude in the object plane A_0 with the Fresnel propagation function h_Z

$$A_Z = A_0 * h_Z = U_{inc}.\alpha_0 * h_Z = U_{inc}.\alpha_Z \qquad (2)$$

$$h_Z(r) = \frac{1}{j\lambda Z}\exp(jkZ)\exp\left(j\frac{\pi r^2}{\lambda Z}\right) \qquad (3)$$

where $k = 2\pi/\lambda$ is the wave number and j is the unit imaginary number such that $j^2 = -1$.

We define α_Z the normalized complex amplitude in the sensor plane, as:

$$\alpha_Z = m_Z \exp(j\phi_Z) \qquad (4)$$

where $\phi_Z = Arg(\alpha_Z)$ is the phase and m_Z is the modulus of α_Z.

The intensity I_Z is recorded by the sensor without any phase information:

$$I_Z = |A_Z|^2 = |U_{inc}|^2 . |\alpha_Z|^2 \qquad (5)$$

$|U_{inc}|^2$ is the background intensity that is recorded when there is no object above the sensor. Note that without object, $\alpha_0 = 1$ and consequently $I_Z = |U_{inc}|^2$. The modulus m_Z is obtained through the measurement of I_Z:

$$m_Z = |\alpha_Z| = \sqrt{\frac{I_Z}{|U_{inc}|^2}} \qquad (6)$$

The purpose of the holographic reconstruction algorithm is to recover the complex image of the sample α_0 from the phase-less recorded holographic image m_Z [11]. We have developed an algorithm based on the formulation of the phase retrieval problem discussed in [19]. After initialization, the algorithm refines the estimate of the complex image in the sensor plane by iteratively applying update rules in a gradient descent. (Step 1) of our phase retrieval algorithm consists in the initialization of the complex amplitude in the sensor plane α_Z by setting its phase to zero: $\phi_Z = 0$. (Step 2) consists in back-propagating the complex amplitudes to the object plane, using the inverse Fresnel function (Eq. 3):

$$\alpha_0 = \alpha_Z * h_{-Z} = m_Z * h - Z \qquad (7)$$

Fig. S5 shows as an example the module and the phase of α_0 after a single back-propagation of lensfree acquisition of a CSF specimen. At this stage the complex image is impaired by the presence of an artefact, the so-called “twin image” that results from a lack of phase information during the acquisition process. In (Step 3) we define a cost function $\varepsilon(\alpha_0)$ to be minimized in order to reduce the “twin image” artefact. Here in the case of CSF sample, we used the total variation norm:

$$\varepsilon(\alpha_0) = \int\int dx.dy.\sqrt{\left|\frac{\partial \alpha_0(x,y)}{\partial x}\right|^2 + \left|\frac{\partial \alpha_0(x,y)}{\partial y}\right|^2} \quad (8)$$

This cost function is minimized when the objects are locally uniform and sparsely distributed which is verified in the case of CSF sample. As the signals are discrete, the derivative operators are replaced by Sobel operators (S_x, S_y) and the integrals by sums. The cost function rewritten as a function of the unknown φ_z of the problem becomes:

$$\begin{aligned} &\varepsilon(\varphi_z) = \sum_{i,j} \sqrt{(S_x * \alpha_0(\varphi_z))_{ij}.(S_x * \alpha_0(\varphi_z))_{ij}^* + (S_y * \alpha_0(\varphi_z))_{ij}.(S_y * \alpha_0(\varphi_z))_{ij}^*} \\ &S_x = \begin{bmatrix} 1 & 0 & -1 \\ 2 & 0 & -2 \\ 1 & 0 & -1 \end{bmatrix}, S_y = \begin{bmatrix} 1 & 2 & 1 \\ 0 & 0 & 0 \\ -1 & -2 & -1 \end{bmatrix} \\ &\alpha_0(\varphi_z) = \left(m_z e^{i\varphi_z}\right) * h_{-z} \end{aligned} \quad (9)$$

where i and j are the indices of pixels. In the following (Step 4) the unknown φ_z is iteratively refined according to a conjugated gradient scheme:

$$\varphi_z^{(k)} = \varphi_z^{(k-1)} + s^{(k)}.p^{(k)} \quad (10)$$

where $p^{(k)}$ is the direction of the descent (with the same dimensions as φ_z) and $s^{(k)}$ is a scalar which gives the step length in the direction of descent. In Eq. (10), the choice of the direction of descent $p^{(k)}$ is given as a function of the gradient of the cost function $\varepsilon(\varphi)$ and the direction obtained at the previous iteration :

$$p^{(k)} = -\nabla\varepsilon + \beta p^{(k-1)} \quad (11)$$

with $p^{(-1)} = 0$ and β a scalar defined according to e.g. the Fletcher-Reeves method :

$$\beta^{(k)} = \frac{\nabla\varepsilon^{(k)}.\nabla\varepsilon^{(k)}}{\nabla\varepsilon^{(k-1)}.\nabla\varepsilon^{(k-1)}} \qquad (12)$$

The gradient of the cost function, e.g. the variation of the cost function for every variation in φ_Z is obtained analytically from Eq. 9 :

$$\nabla\varepsilon = \frac{\partial\varepsilon}{\partial\varphi_Z} = \operatorname{Im} A_Z^* \left(\left(S_x * \frac{S_x * A_0}{\sqrt{\left|S_x * A_0\right|^2 + \left|S_y * A_0\right|^2}} + S_y * \frac{S_y * A_0}{\sqrt{\left|S_x * A_0\right|^2 + \left|S_y * A_0\right|^2}} \right) * h_Z \right) \qquad (13)$$

In Eq. (10), the step length $s^{(k)}$ is given by a minimization-majoration method [20] obtained by majoring the cost function $\varepsilon(\varphi_Z)$ (Eq. (9)) by a quadratic form:

$$s^{(k)} = -\frac{\operatorname{Re}\sum_{ij}\left(X_{ij}\Delta X_{ij}^* + Y_{ij}\Delta Y_{ij}^* \right)}{\sum_{ij}\left(\Delta X_{ij}\Delta X_{ij}^* + \Delta Y_{ij}\Delta Y_{ij}^* \right)}$$

$$D_{ij} = \sqrt{\left(S_x * \alpha_0\left(\varphi^{(k-1)} \right) \right)_{ij} . \left(S_x * \alpha_0\left(\varphi^{(k-1)} \right) \right)_{ij}^* + \left(S_y * \alpha_0\left(\varphi^{(k-1)} \right) \right)_{ij} . \left(S_y * \alpha_0\left(\varphi^{(k-1)} \right) \right)_{ij}^*}$$

$$X_{ij} = \left(S_x * \alpha_0\left(\varphi^{(k-1)} \right) \right)_{ij} / D_{ij}$$

$$Y_{ij} = \left(S_y * \alpha_0\left(\varphi^{(k-1)} \right) \right)_{ij} / D_{ij}$$

$$\Delta X_{ij} = i\left(S_x * pA_Z\left(\varphi^{(k-1)} \right) * h_{-Z} \right)_{ij} / D_{ij}$$

$$\Delta Y_{ij} = i\left(S_y * pA_Z\left(\varphi^{(k-1)} \right) * h_{-Z} \right)_{ij} / D_{ij}$$

(14)

(Step 4) is repeated typically 30 times to obtain the convergence of our algorithm.

Measurement consistency is ensured since module m_Z is kept unchanged all along the

iterative process (Eq. (9). Finally, the reconstructed complex image α_0 is obtained by propagating the refined complex image α_Z to the object plane (Fig. S5).

SUPPLEMENTARY FIGURE LEGENDS

Figure S1. Comparison between (a) the transmission image obtained with x10 magnification microscope and (b) the lensfree RGB reconstructed module image. Red and blue circles denote respectively erythrocytes (RBCs) and leukocytes (WBCs) as confirmed on (a) the microscope transmission image. The reconstructed (red channel) phase and module Z-axis profiles corresponding to the different cells circled in (a) are shown in (c) and (d) respectively. The leukocytes profiles are plotted with dotted lines and the erythrocytes profiles with plain line. The reconstructed phase and module Z-axis profiles (blue channel) are shown i n (e) and (f) respectively.

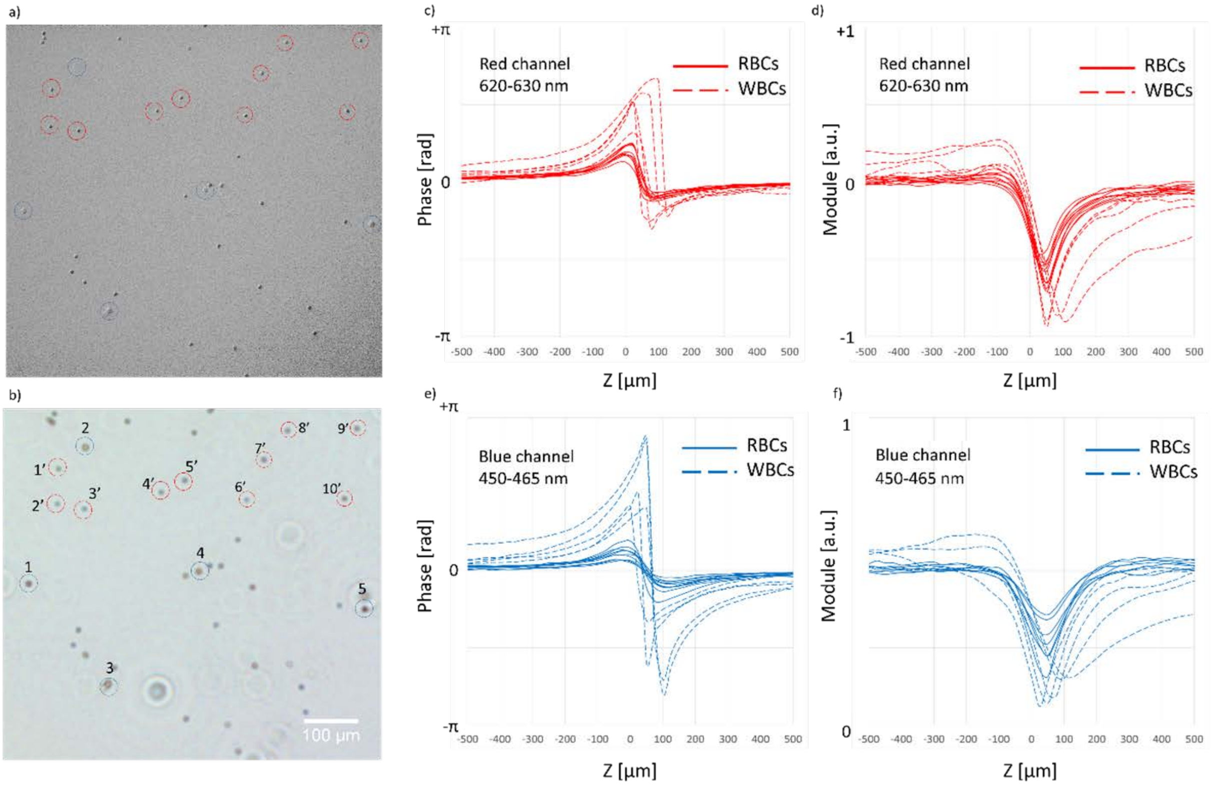

Figure. S2. Comparison between the lensfree automatic count and the reference optic microscopy counting for 215 cerebrospinal fluid clinical specimens (a) leukocytes counts (b) erythrocyte counts.

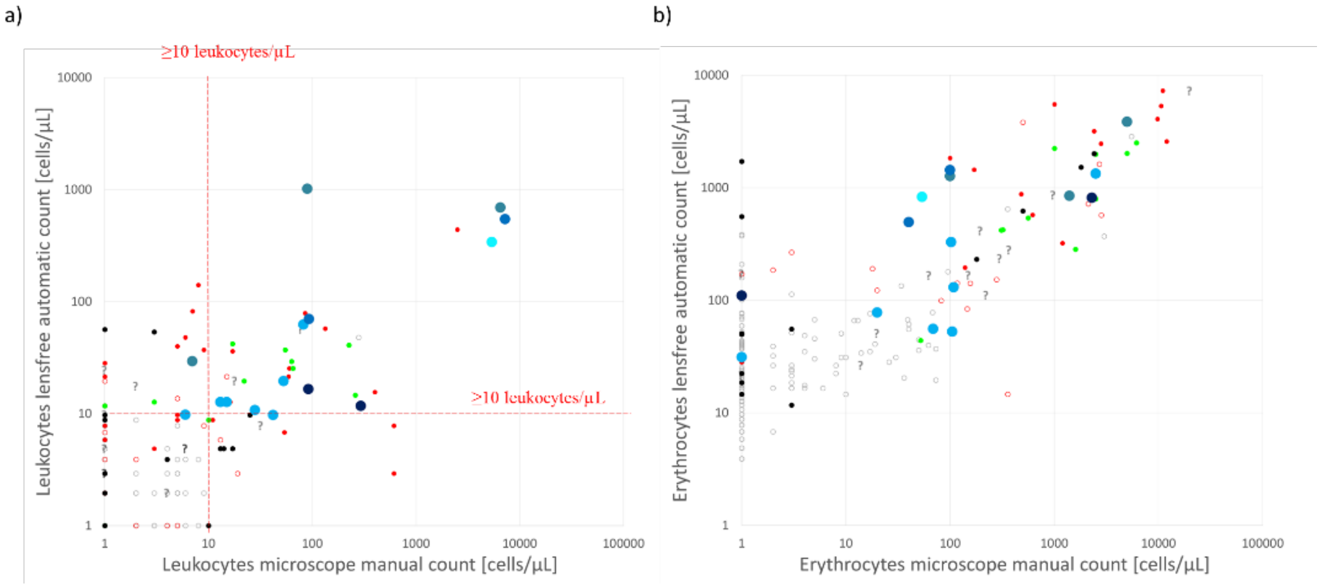

Figure S3. Scatterplot of the optic microscopy leukocyte and erythrocyte counts resulting from the analysis of the first datasets featuring 215 clinical specimens. A color code has been defined with respect to the different diagnosis established for all clinical specimens. The infectious meningitis of interest are plotted in large blue dots. The limit of 10 cells/µL used for the biological definition of meningitis is depicted by a horizontal red dotted line. The obtained sensitivity is about 87% and the specificity is about 85%. There are two cases two cases of infectious meningitis which have not been detected under microscope, the leukocytes microscope count was respectively 6 and 7 leukocytes/µL well below the limit of 10 cells/µl used for the early diagnoses of meningitis. The specificity can be improved up to 87% if additional criteria are applied to detect hemorrhagic samples in which the number of erythrocyte is > 10.000 cells/µL.

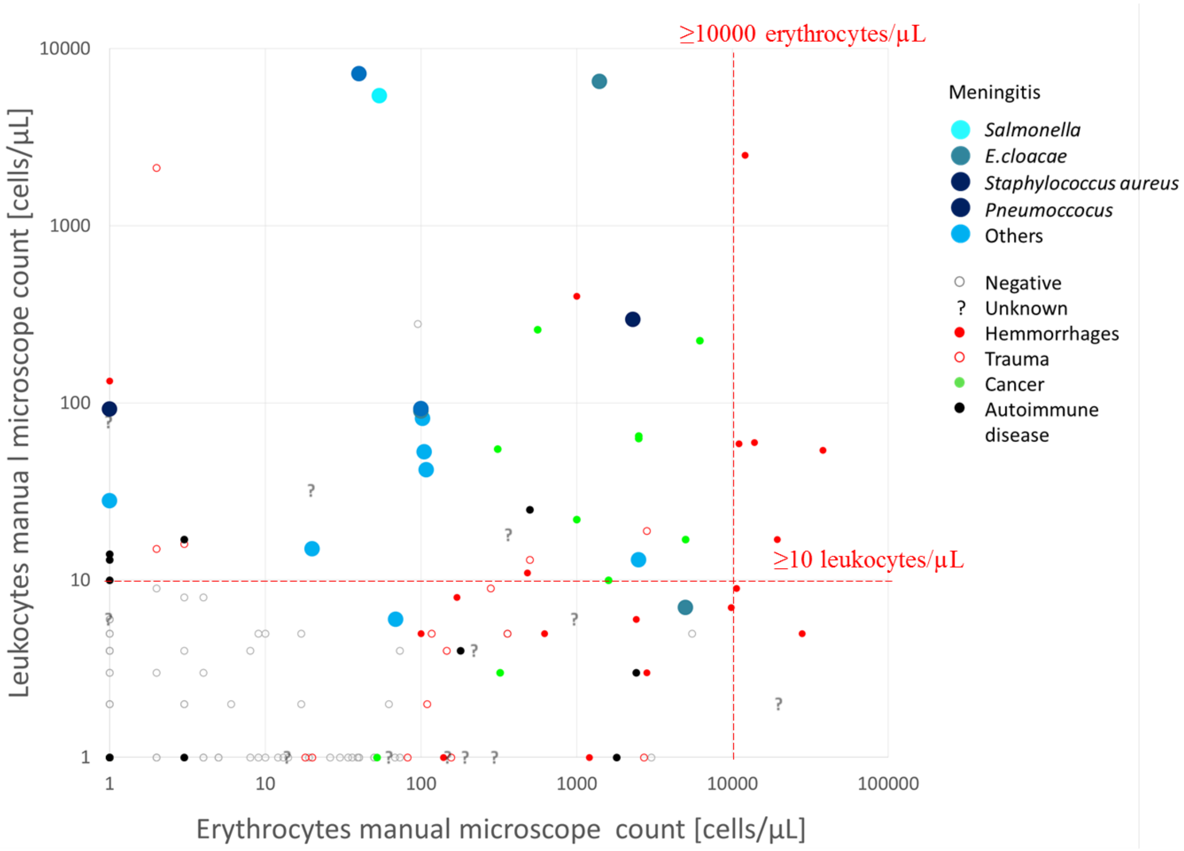

Figure S4. Principle of lens-free in-line holography. The object is located at z=0, the sensor is at *z*=*Z*. Radial coordinates are defined by the vector $\vec{r}$. The object is illuminated by an incident plane wave U_{inc} . α_0 is the normalized complex transmission of the object. α_Z is the normalized complex amplitude at the sensor plane.

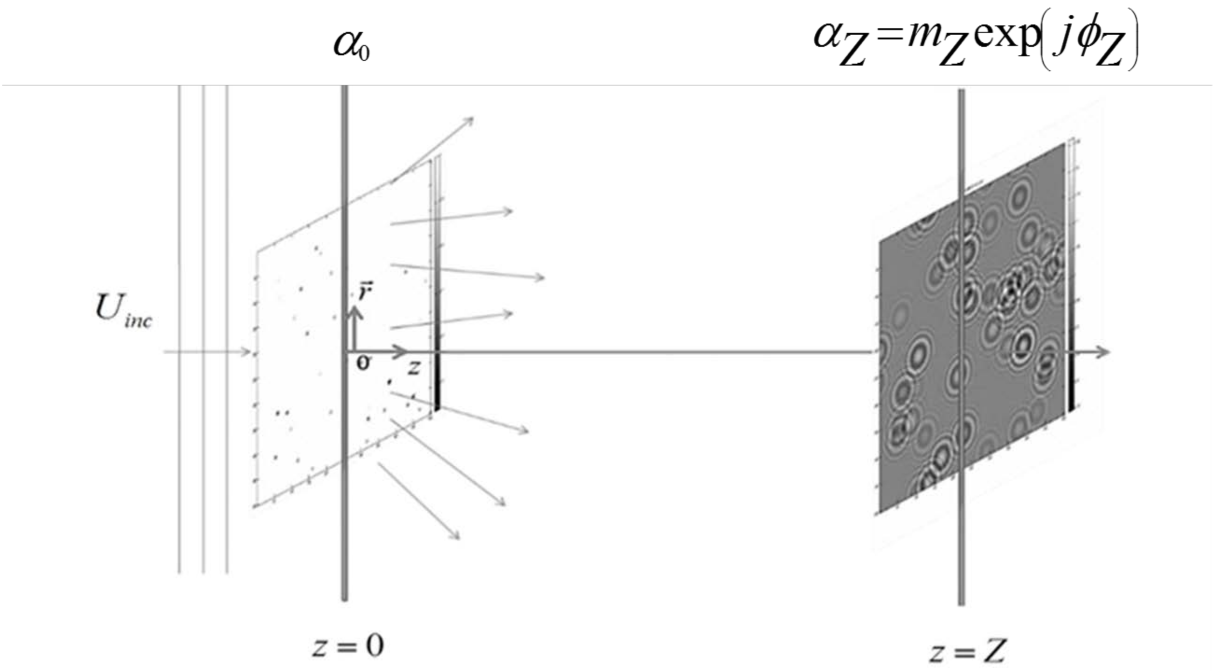

Figure S5. (a) Lensfree raw hologram acquired in the blue channel of the sample Q160550287 (ruptured brain aneurysm; microscope counting: 218 erythrocytes/µL, 14 leukocytes/µL). (b) Reconstructed module image after a single back-propagation. The image shows the presence of the so-called ‘twin image’, the cells are surrounded by concentric rings which result of lack of phase information. (c) Reconstructed module image obtained after 30 iterations of the phase-retrieval algorithm. The phase in the sensor plane is well estimated and the reconstructed image is now free of ‘twin image’ (d) Reconstructed phase image after a single back-propagation. (e) Reconstructed phase image obtained after 30 iterations of the phase-retrieval algorithm. All images are cropped area of 0.65µm^2 out of the full field of view of 29.4mm^2.

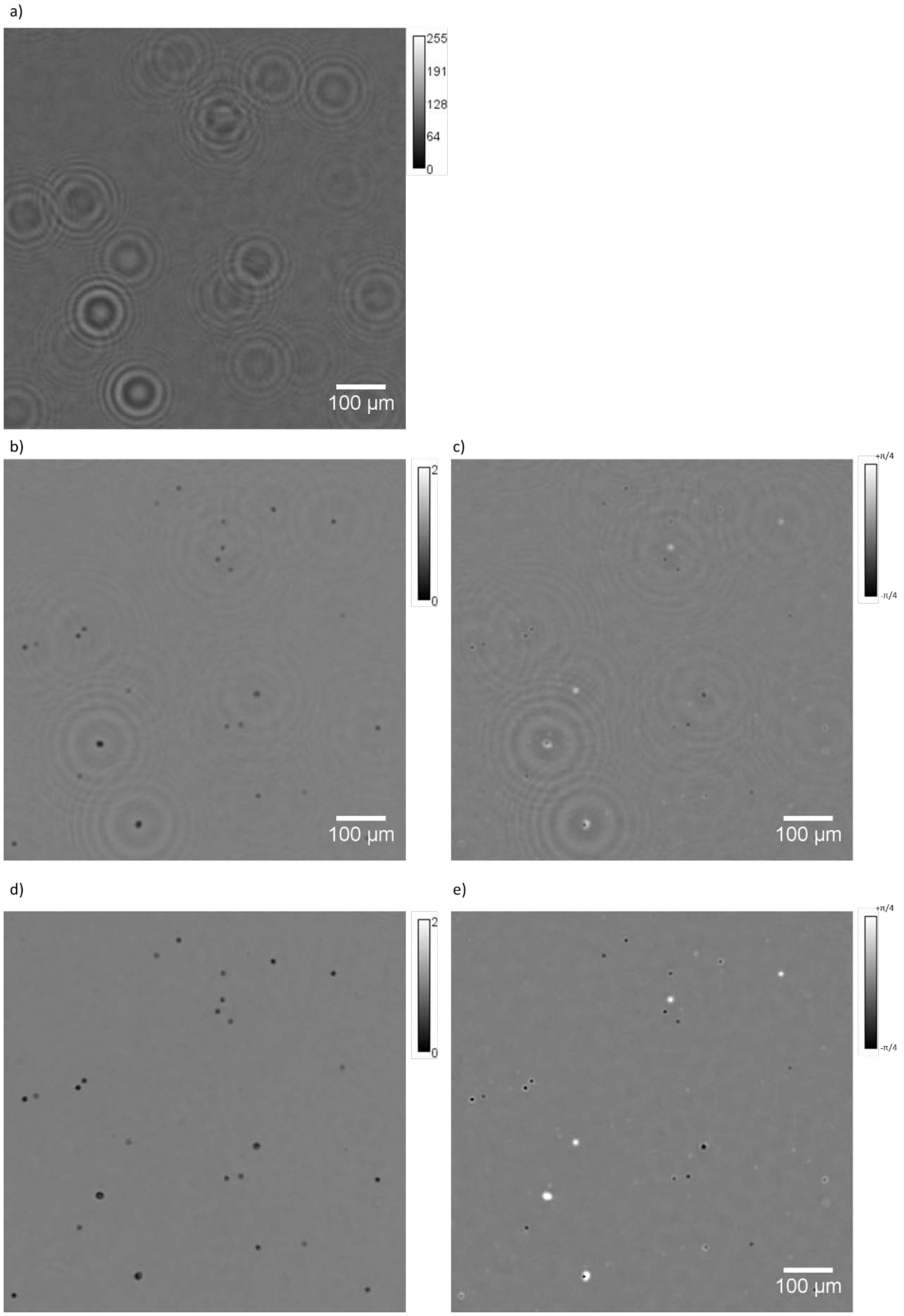
a)
255
191
128
64
0
100 µm
b)
2
0
100 µm
c)
+π/4
-π/4
100 µm
d)
2
0
e)
+π/4
-π/4
100 µm

Figure S6. (a) Cell classification performed on the module reconstructed image (red channel) of the sample Q150430694 (autoimmune disease; microscope counting: 1800 erythrocytes/µL, 0 leukocytes/µL) over a cropped area of 11.6µm^2. Red circles denote the detection of the erythrocytes, black circles denote the detection of small particles or false detections, and blue circles denote objects with large phase amplitude which are likely leukocytes (b) Detail of (a) (yellow rectangle).

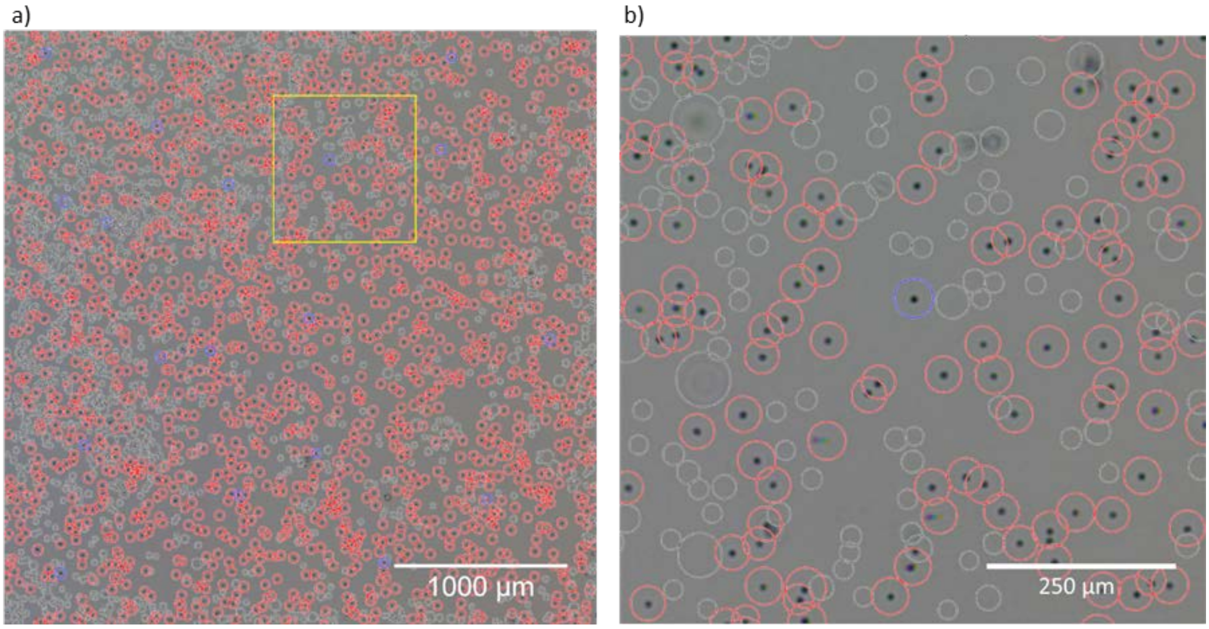

Supplement: Supplementary Information [file srep39893-s1.pdf]
