# Supplementary material for: Cerebrospinal fluid lens-free microscopy: a new tool for the laboratory diagnosis of meningitis
Source: Sci Rep. 2017 Jan 3;7:39893. doi: 10.1038/srep39893 (PMC5206666; doi:10.1038/srep39893)
Supplement: Supplementary Table 1 [file srep39893-s2.pdf]

CSF	Operator 1	Operator 2	Operator 3	Operator 4	Operator 5
1 WC	3	3	0	1	5
1 RC	14	10	0	7	13
2 WC	0	0	1	0	0
2 RC	0	1	0	0	0
3 WC	1	0	0	0	0
3 RC	2	0	2	4	1
4 RC	0	0	0	2	0
4 RC	1500	1400	800	2000	1500
5 WC	0	0	0	1	1
5 RC	6	3	1	5	4
6 WC	12	7	5	12	9
6 RC	200	150	300	350	350
7 WC	0	0	0	0	0
7 RC	0	1	1	0	0
8 WC	5	3	31	3	6
8 RC	10	10	17	9	13
9 WC	90	62	95	74	87
9 RC	1500	1000	1200	1400	1500
10 WC	1	0	1	1	1
10 RC	1	1	2	5	3
1 WC	5	5	6	7	15
1 RC	5	6	7	8	23
2 WC	15	20	20	25	25
2 RC	3	4	4	5	4
3 WC	7	8	9	11	9
3 RC	5	6	7	8	8
4 WC	5	6	7	7	7
4 RC	40	50	63	62	50
5 WC	10	13	20	18	16
5 RC	1	3	2	1	2
6 WC	0	0	0	0	0
6 RC	10000	10000	10000	10000	10000
7 WC	5	6	6	7	5
7 RC	10	12	13	10	20
8 WC	0	0	0	0	0
8 RC	0	0	0	0	0
9 WC	100	130	122	131	117
9 RC	10000	10000	10000	10000	10000
10 WC	0	0	0	0	1
10 RC	35	40	36	40	36
1 WC	239	280	292	275	303
1 RC	833	750	875	700	889
2 WC	0	1	0	1	0
2 RC	0	1	0	1	0
3 WC	7	4	4	5	5
3 RC	9	12	11	10	10
4 WC	0	0	43	0	0
4 RC	10000	10000	10000	10000	10000

5 WC	2	3	2	2	3
5 RC	6	12	10	8	7
6 WC	1	0	0	0	0
6 RC	0	0	3	0	0
7 WC	26	18	40	25	24
7 RC	256	312	420	300	322
8 WC	0	0	0	7	5
8 RC	300	320	324	411	426
9 WC	60	56	52	62	100
9 RC	30	33	31	30	45
10 WC	0	1	0	1	1
10 RC	0	0	0	0	0
1 WC	10	15	12	22	18
1 RC	30	32	44	51	50
2 WC	1	0	2	0	10
2 RC	35	40	42	30	31
3 WC	8	2	0	9	8
3 RC	148	245	200	310	200
4 WC	1	2	1	9	4
4 RC	1600	1200	2000	2584	1586
5 WC	0	0	0	0	0
5 RC	2	2	3	2	2
6 WC	2	2	3	8	1
6 RC	5	5	7	15	2

Supplementary Table 1. Interoperator (5 operators) variability in cerebrospinal fluid cell counting by standard optic microscopy.

CSF: cerebrospinal fluid

RC: Red cells

WC: White cells
